# Influence of environmental and anthropogenic acoustic cues in sea-finding of hatchling leatherback (*Dermochelys coriacea*) sea turtles

**DOI:** 10.1371/journal.pone.0253770

**Published:** 2021-07-01

**Authors:** Bethany Holtz, Kelly R. Stewart, Wendy E. D. Piniak

**Affiliations:** 1 Department of Environmental Studies, Gettysburg College, Gettysburg, Pennsylvania, United States of America; 2 The Ocean Foundation, Washington, D.C., United States of America; Wildlife Conservation Society Canada, CANADA

## Abstract

Although the visual and geomagnetic orientation cues used by sea turtle hatchlings during sea-finding have been well studied, the potential for auditory stimuli to act as an orientation cue has not been explored. We investigated the response of sea turtle hatchlings to natural and anthropogenic noises present on their nesting beaches during sea-finding. The responses of hatchling leatherback sea turtles, *Dermochelys coriacea*, collected from the Sandy Point National Wildlife Refuge, St. Croix, were measured in the presence of aerial acoustic sounds within hatchlings’ hearing range of 50 to 1600 Hz. The highest sound energy produced by beach waves occurs at frequencies 50–1000 Hz, which overlaps with the most sensitive hearing range of hatchling leatherbacks (50–400 Hz). Natural beach wave sounds, which have highest sound energy at frequencies of 50–1000 Hz, may be masked by human conversations (85–650 Hz) and vehicle traffic noise (60–8000 Hz). In the presence of three stimuli, a) beach wave sounds (72.0 dB re: 20 μPa), b) human conversation (72.4 dB re: 20 μPa), and c) vehicle traffic noise (71.1 dB re: 20 μPa), hatchlings exhibited no phonotaxic response (wave sounds: mean angle = 152.1°, p = 0.645; human conversation: mean angle = 67.4°, p = 0.554; traffic noise: mean angle = 125.7°, p = 0.887). These results may be due to the hatchlings being unable to localize sounds in the experimental arena. Visual and auditory cues may also converge to affect sea-finding orientation. Future studies should focus on the localization ability of sea turtles and on the role that sound may play in orientation when combined with other sensory and environmental cues.

## Introduction

Increased levels of anthropogenic sound in the marine environment have been a focus of marine physiology, ecology, and conservation research over the last several decades [1–6]. Relationships between these elevated sound levels and stress, behavioral responses, and threshold shifts in hearing have already been noted in several species of marine mammals and fishes [2, 5–10]. However, research on the impacts of anthropogenic sound on sea turtles has lagged behind, with only a few studies examining the behavioral responses of sea turtles to sound [11–13]. As both the frequency and range of anthropogenic activities responsible for these sounds intensify, studies examining the ways in which marine organisms respond to these non-natural auditory stimuli will continue to be of keen importance. Studies examining the potential behavioral and physiological impacts of noise on sea turtles are needed [14].

Sea turtles hear through a vertebrate tympanic middle ear path: a tympanum connected to facial tissue, an air-filled middle ear cavity, and a connection to the inner ear via a single middle ear bone [15–17]. Studies measuring the hearing range of juvenile green (*Chelonia mydas*), hatchling hawksbill (*Eretmochelys imbricata*), and hatchling leatherback sea turtles (*Dermochelys coriacea)* in both air and water revealed that sea turtles can detect low-frequency aerial and underwater acoustic signals between 50–1600 Hz with maximum sensitivity between 100–400 Hz [11, 14, 18]. For all three species, when aerial and underwater hearing thresholds are compared in terms of pressure, thresholds for hearing in air are lower than those in water [11, 14, 18]. However, when hearing thresholds are compared in terms of sound intensity, these species are more sensitive (have lower thresholds) to underwater stimuli [11, 14, 18]. Low-frequency anthropogenic sounds overlapping the frequencies sea turtles can detect include sounds produced by airplanes, sonar, shipping, oil and gas exploration and extraction, vehicle traffic, human conversation, and other anthropogenic sources [1, 11, 19–24]. Environmental acoustic sounds within these frequency ranges include the sounds of crashing beach wave sounds and predator vocalizations [1].

While our understanding of sea turtle detection and response to sound has increased greatly over the last decade, the biological significance of sound for sea turtles remains mostly unknown. It is hypothesized that turtles may use sound in navigation, prey location, predator detection and avoidance, and for general environmental awareness [14, 25, 26]. Most studies to date examining the physiological and behavioral impacts of sound have been limited to visual observations of behavioral responses to underwater explosions and seismic airguns by sea turtles [27–32]. In the presence of both explosions and seismic airguns, sea turtles exhibited notable behavioral responses, including erratic swimming and diving behavior, indicating sensitivity to changes in sound pressure [27–32].

As beach environments play a pivotal role in the sea turtle life cycle, and are often impacted by anthropogenic activities, examining how sea turtles may use natural beach sounds as sensory cues is of significance for conservation efforts. Natural sensory cues on the beach can impact survival of hatchlings, which rely heavily upon environmental signals or cues to orient toward and find the ocean [33]. While the visual and slope orientation cues used by hatchlings during sea-finding as they move from the nest to the sea have been well studied [34, 35], the potential for auditory stimuli to act as an orientation cue has not been sufficiently explored. The sounds of beach waves may act as an auditory cue for sea-finding. Hatchling leatherback sea turtles are able to detect aerial acoustic sounds between 50 and 1600 Hz and are most sensitive to sounds between 50 and 400 Hz [11, 14, 18]. The highest sound energy of beach waves occurring at <1000 Hz overlaps with the most sensitive hearing range of hatchling leatherbacks. Also falling within this peak hearing range are human conversation (120–1500 Hz) and vehicle traffic (50–8000 Hz), which may mask auditory orientation or other acoustic cues [21, 23].

As sea turtles are threatened or endangered, reducing their mortality is important for their preservation. Since hatchlings on the nesting beach are often the most accessible age-class, understanding the impacts of how hatchlings respond to environmental and anthropogenic noise along beaches can assist in reducing hatchling mortality. To understand the implications of anthropogenic noise on sea turtles, we must first understand how they use natural sounds as cues. As studies examining sea turtle behavioral responses to sound have been limited, and studies exploring responses to natural environmental acoustic cues are completely lacking, this study aimed to investigate how sea turtles might use acoustic stimuli found in their environment. Specifically, this study explores the potential for natural beach sounds (waves breaking) to act as an orientation cue for hatchlings during sea-finding and also examines the influence of anthropogenic sounds that may be present in the beach environment (human conversation and vehicle traffic).

## Methods

### Sea turtle hatchlings

**Fig 1:**
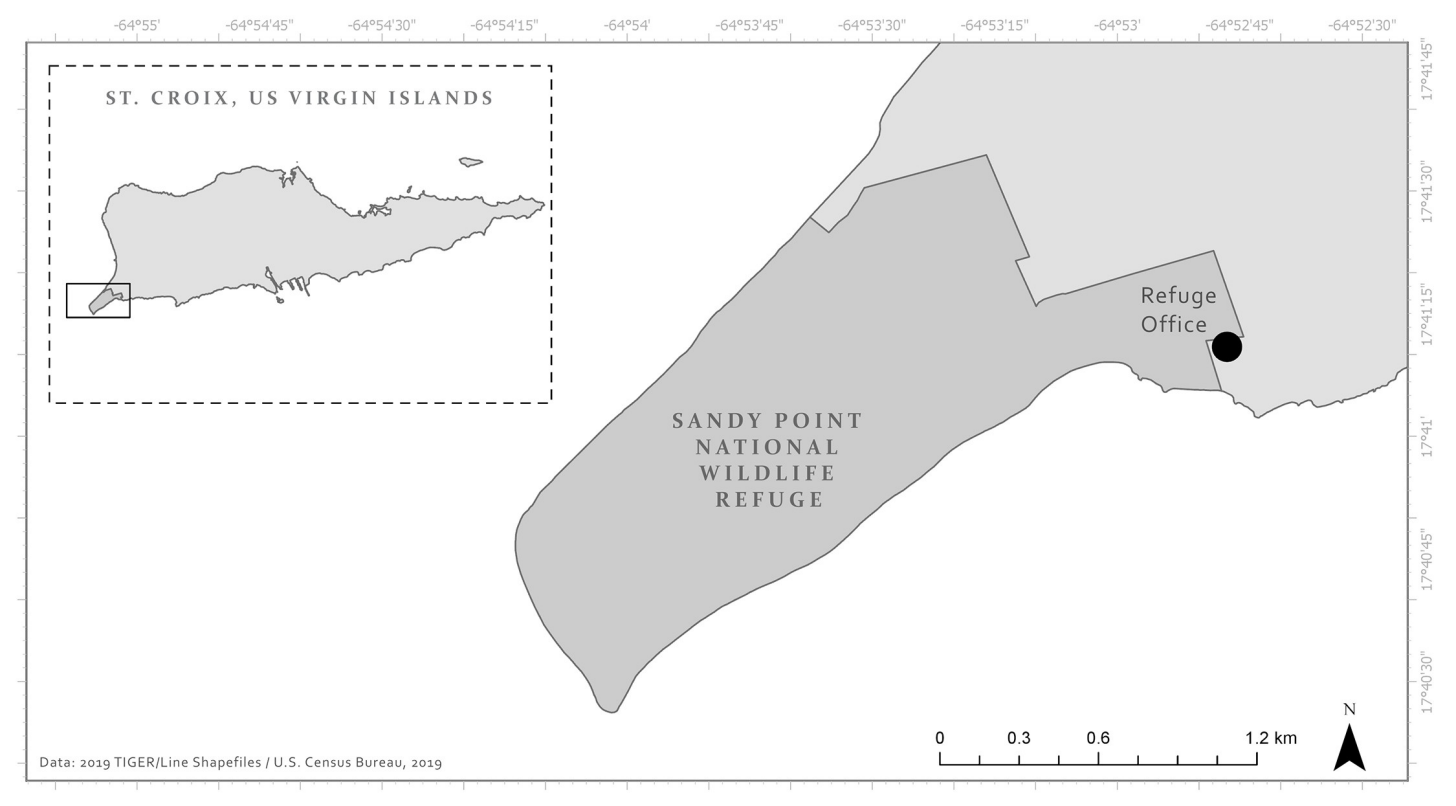
Hatchlings were collected from the beach along the Sandy Point National Wildlife Refuge shoreline as they emerged from their nests. The trials were conducted at the refuge office and hatchlings were returned to the beach for release following testing.

Hatchling leatherback sea turtles were collected from the nesting beach at the Sandy Point National Wildlife Refuge, located at the southwestern tip of St. Croix in the U.S. Virgin Islands (Fig 1). We collected turtles as they emerged naturally from their nests between 5:00 and 8:00 PM and immediately placed into dark buckets to limit visual cues before being transferred to the testing environment within the refuge office. Hatchlings averaged 44.0 ± 2.7 g in weight (range: 36–51.0 g), 63.0 ± 2.5 mm in curved carapace length (range: 47–70.0 mm), 53.0 ± 2.4 mm in curved carapace width (range: 46–64.0 mm), 58.7 ± 2.2 mm in straight carapace length (range: 51–64.4 mm) and 38.9 ± 1.9 mm in straight carapace width (range: 33.0–45.0 mm). All hatchlings were tested and released on the same night of collection.

### Sound recording and generation

#### Beach wave sounds

**Fig 2:**
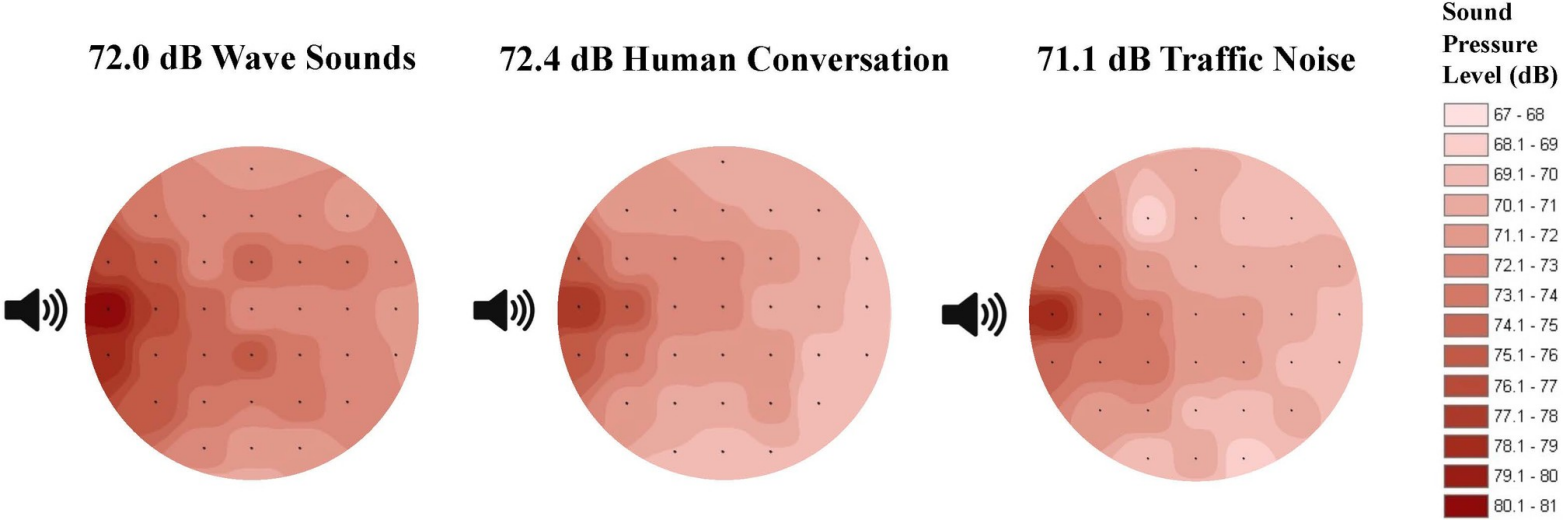
Circular testing arena sound field in dB re: 20 μPa. Average RMS sound pressure levels were recorded at set points shown as black dots. A kriging analysis in ArcGIS was used to estimate sound pressure levels between points.

We recorded wave sounds during varying tidal, wind, and weather conditions using an Earthwork’s M20BX microphone and a M-Audio MicroTrack II Digital Recorder in front of nests along the north, south, and west sides of the refuge beach. The recording with minimal wind and background noise was used for all trials. We chose an average sound level by examining multiple recordings. The trial sound was played at 72.0 dB re: 20μPa (1-18kHz) measured at the center of the arena. The sound pressure level in the leatherback hatchling hearing range (<1600 Hz) was 71.8 dB re: 20 μPa measured at the center of the arena. The recording was 1 minute in length and played on a loop for each 5-minute trial. The signal was played to the hatchlings using a Tascam DR-05 Digital Recorder, and a Definitive Technology DI 6.5R speaker amplified by a Samson Servo 120A amplifier. Using the microphone and M-Audio recorder, we measured the sound at several locations within the arena and used ArcGIS (kriging analysis) to map the testing arena’s sound field to determine the sound level perceived by hatchlings at locations throughout the testing arena for each auditory signal (Fig 2).

#### Anthropogenic sound

Recordings for human conversation were collected along the nesting beach using the microphone and digital recorder described above. A compilation of these recordings with various people and number of people talking was used as the trial signal. We chose an average sound level of 72.4 dB re: 20μPa (1–20 kHz) measured at the center of the arena. The sound pressure level in the leatherback hatchling hearing range (<1600 Hz), was 71.4 dB re: 20 μPa measured at the center of the arena. We recorded vehicle traffic during various traffic patterns on a road neighboring the nesting beach from which traffic noise could still be heard on the beachfront. We chose an average sound level of 71.1 dB re: 20 μPa (1–18 kHz) measured at the center of the arena for use as the experimental level. The vehicle traffic noise file with minimal background and wind noise was used for experimentation. The sound pressure level in the leatherback hatchling hearing range (<1600 Hz) was 70.9 dB re: 20 μPa measured at the center of the arena. Both the human conversation and vehicle traffic sounds were played to the hatchlings using the same equipment mentioned above for the beach wave trials using a 1 minute recording played on a loop for each 5 minute trial. We created a map of the sound field for both human conversation and vehicle traffic using the methodology previously mentioned (Fig 2).

#### Background noise

Efforts to minimize background noise in the refuge office were undertaken during arena construction and during the trials, including turning off electronics, air conditioning, and overhead lighting. The refuge office is distant from busy roads and located far enough from the beach that wave sounds are not audible inside the building. Background noise in the refuge office was 54 dB dB re: 20 μPa. Trial sounds were played at 72.0, 71.1 or 72.4 dB re: 20 μPa measured at the center of the arena. This ensured that trial sounds throughout the testing arena were greater than 10 dB louder than the background noise.

### Sound orientation experimental design

**Fig 3:**
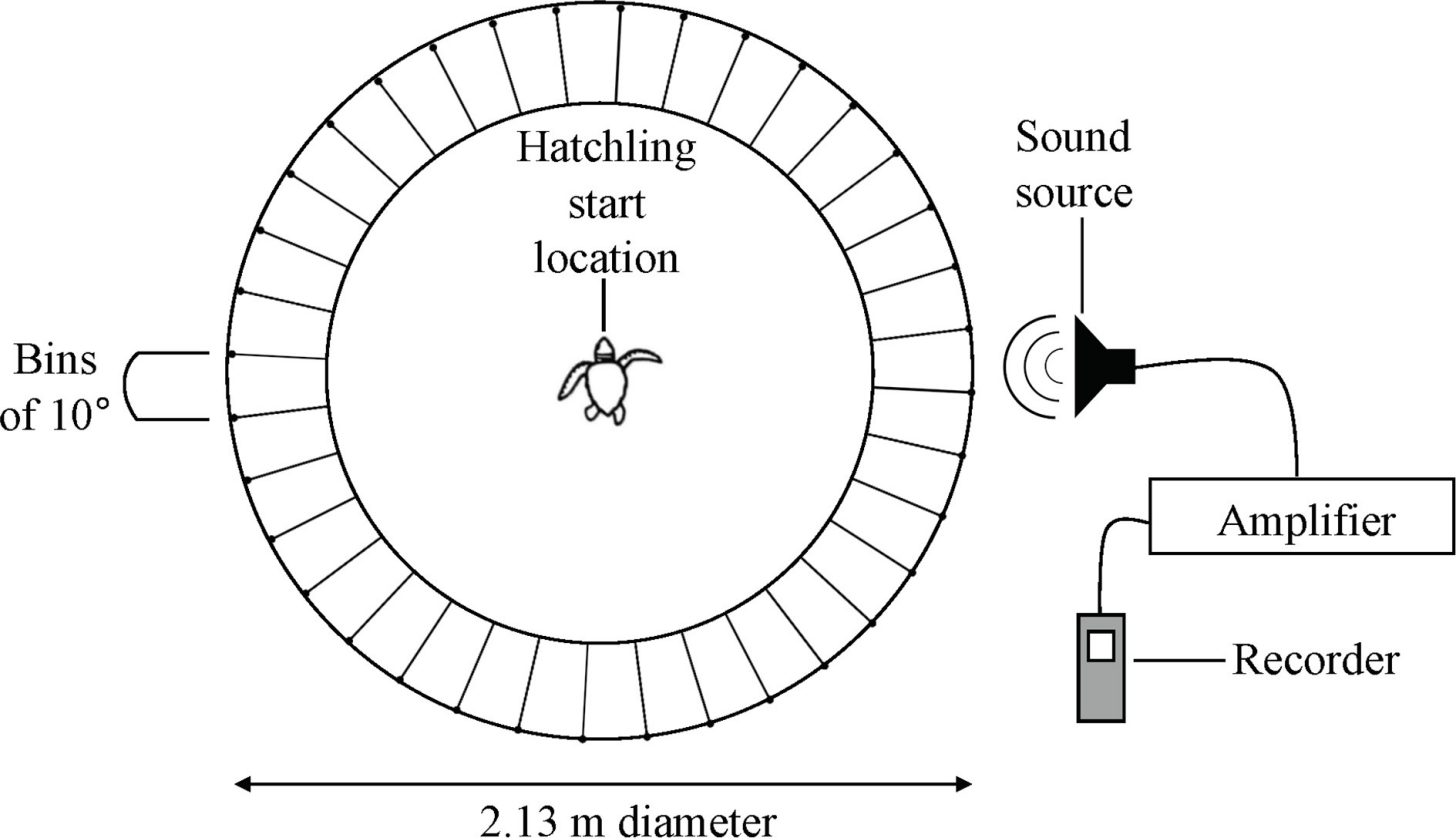
Circular arena surrounded by 36 bins. The arena was encased in a light-proof tent and a speaker producing the trial sounds was rotated outside the tent at four alternating cardinal directions.

We examined hatchling response to trial auditory signals in a circular arena raised 14.0 cm off the floor. The arena’s circular platform was surrounded by 36 converging cushioned pitfall bins constructed to correspond to 10° intervals (Fig 3). We used a compass to determine the degree increments along the edge of the circular platform and the outer bins of the platform were then constructed to align with these measurements. We encased the entire arena in a light-proof tent to eliminate light cues. A speaker producing the recorded beach wave and anthropogenic sounds was placed outside the tent and moved to alternating locations (in relation to compass bearings: North, South, East, and West) to ensure turtles were orienting toward the speaker and not in relation to any given compass bearing. We suspended the speaker producing the trial sounds from a PVC frame above the table and off the floor to prevent vibrations. We leveled the platform to eliminate potential slope cues.

We placed a single hatchling into the middle of the arena and gave the hatchling five minutes to orient/crawl into a bin. Testing consisted of two phases with phase two contingent on the success of phase one in order to minimize the number of hatchlings tested. An N size of 40 successful trials was set for each trial group, where a successful trial was defined as one in which the hatchling oriented into a bin within the allotted time. In trial groups where an unsuccessful trial occurred, additional trials were conducted. Prior to phase one, the first 10 hatchlings collected were exposed to no sound to serve as a pre-test and ensure all equipment was working properly. We randomly assigned hatchlings to an experimental trial or control from each nest that emerged on a given night to increase the variation in the sample. In phase one, we exposed hatchlings to either beach wave sounds or a control consisting of no sound. Once it was confirmed that hatchlings moved in the testing arena and crawled into a bin within the allotted trial time, phase two was initiated. In phase two, we exposed hatchlings to either human conversation, vehicle traffic, or a control of no sound. A Lorex DV800 Series infra-red camera system was used to observe the time elapsed and note the final bin location of the hatchlings from outside of the tent to prevent human interference. A total of 173 turtles were tested, with 10 hatchlings in the pre-test group, 41 hatchlings in the control group, 40 hatchlings in the wave sounds group, 41 hatchlings in the human conversation group, and 41 hatchlings in the vehicle traffic group. Each hatchling participated in only one trial and were only exposed to one sound type or to a control of no sound.

### Statistical analysis

Circular statistics were used to determine the orientation of hatchlings in each trial group relative to speaker location. Each bin had a degree range of 10° and the midpoint of this range was used for statistical analysis. For example, if a hatchling crawled into bin 1 they were deemed to be orienting between 0-10° and 5° was the value used for analysis. We used NCSS Statistical Software to calculate the mean orientation angle of each trial group and generate rose plots to depict the final orientation angle of each hatchling relative to other hatchlings within each grouping. Oriana was utilized to run a Rayleigh test to test whether the observed angles had a tendency to cluster around or away from the trial sounds. As the cardinal direction of the speaker and the potential magnetic field of the speaker itself were determined to have no effect, speaker location was corrected so that the speaker would always be represented at 0° for each trial (S1 Table).

We conducted an independent T-test to measure if there was a significant difference in travel time between the control group and each of the three experimental treatment groups. The Aspen-Welch Unequal-Variance T-Test and Equal-Variance T-Test were used respectively if we failed to reject the null hypotheses, which assumed the two samples had equal variance and were normally distributed.

### Ethics statement

Sea turtle hatchlings were collected from the nesting beach at the Sandy Point National Wildlife Refuge and returned to the nesting beach for release the same night of collection. This study was approved by the Gettysburg College Institutional Animal Care and Use Committee (Protocol #2015F1), the U.S. Fish and Wildlife Service (Permit #2018–001), and the Department of Planning and Natural Resources Division of Fish and Wildlife (Permit #DFW18058X).

## Results

**Fig 4:**
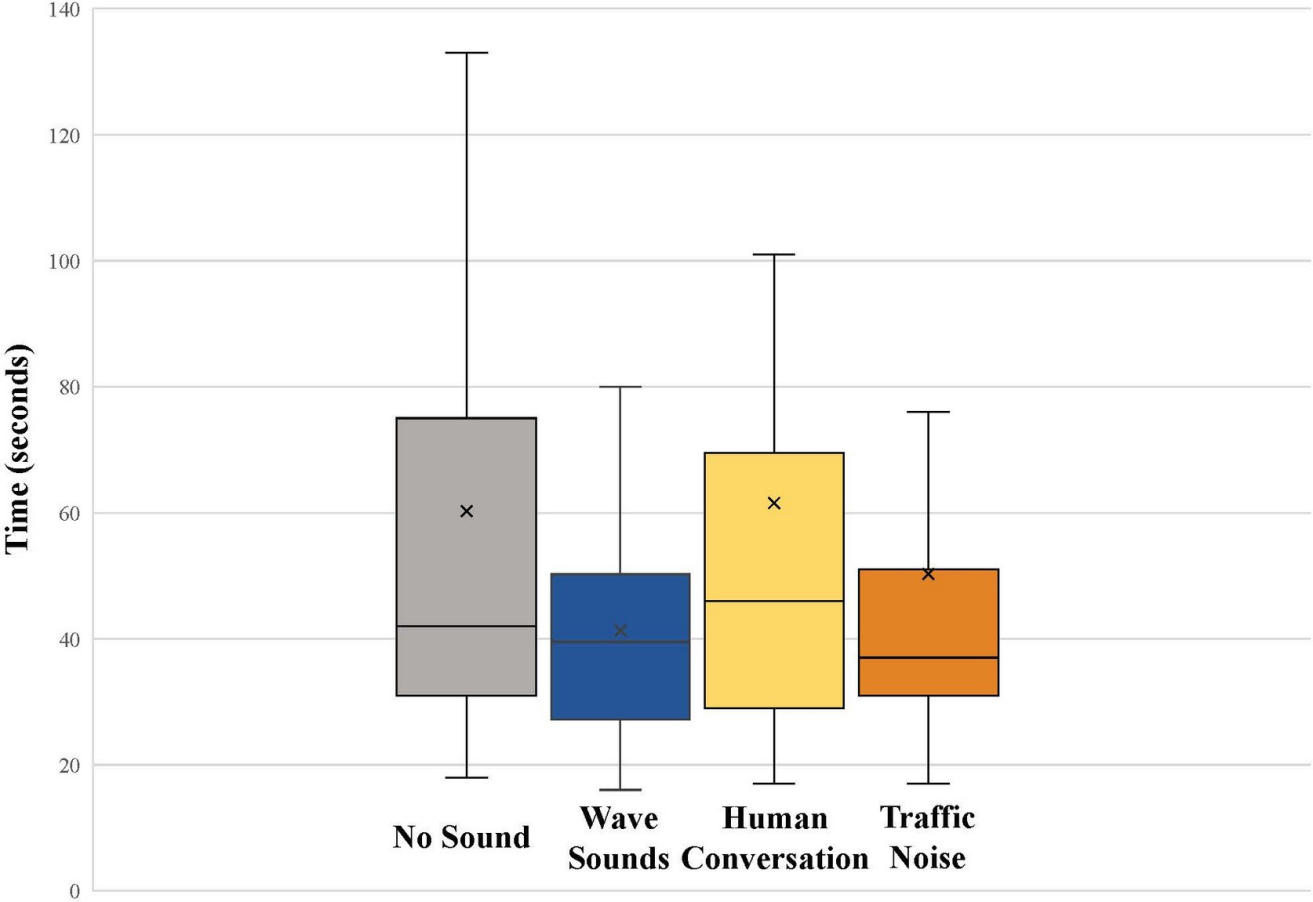
Time spent in the circular arena per trial group. Time began when the hatchling was placed in the arena and ended when they crawled into one of the 36 converging bins surrounding the central circular platform. The x within each plot represents the mean. (No Sound: N = 41, Wave Sounds: N = 40, Human Conversation: N = 41, Traffic Noise: N = 41).

Of the 173 hatchlings tested in the orientation trials, only 3 turtles failed to orient within the circular arena in the 5-minute allotted trial time. Hatchlings that did orient or reach a final bin location within the arena did so in an average of 53 ± 47 seconds (range: 16–300 seconds), with 71% orienting in less than one minute and 89% orienting in under two minutes. Within the control trial, turtles oriented in 60 ± 50 seconds (range: 18–300 seconds). In the wave sounds trial group, hatchlings oriented in 41 ± 19 seconds (range: 16–101 seconds). Hatchlings in the human conversation trial group oriented in 62 ± 61 seconds (range: 17–300 seconds), while those in the traffic noise trial group oriented in 50 ± 47 (range: 17–300 seconds) (Fig 4).

**Fig 5:**
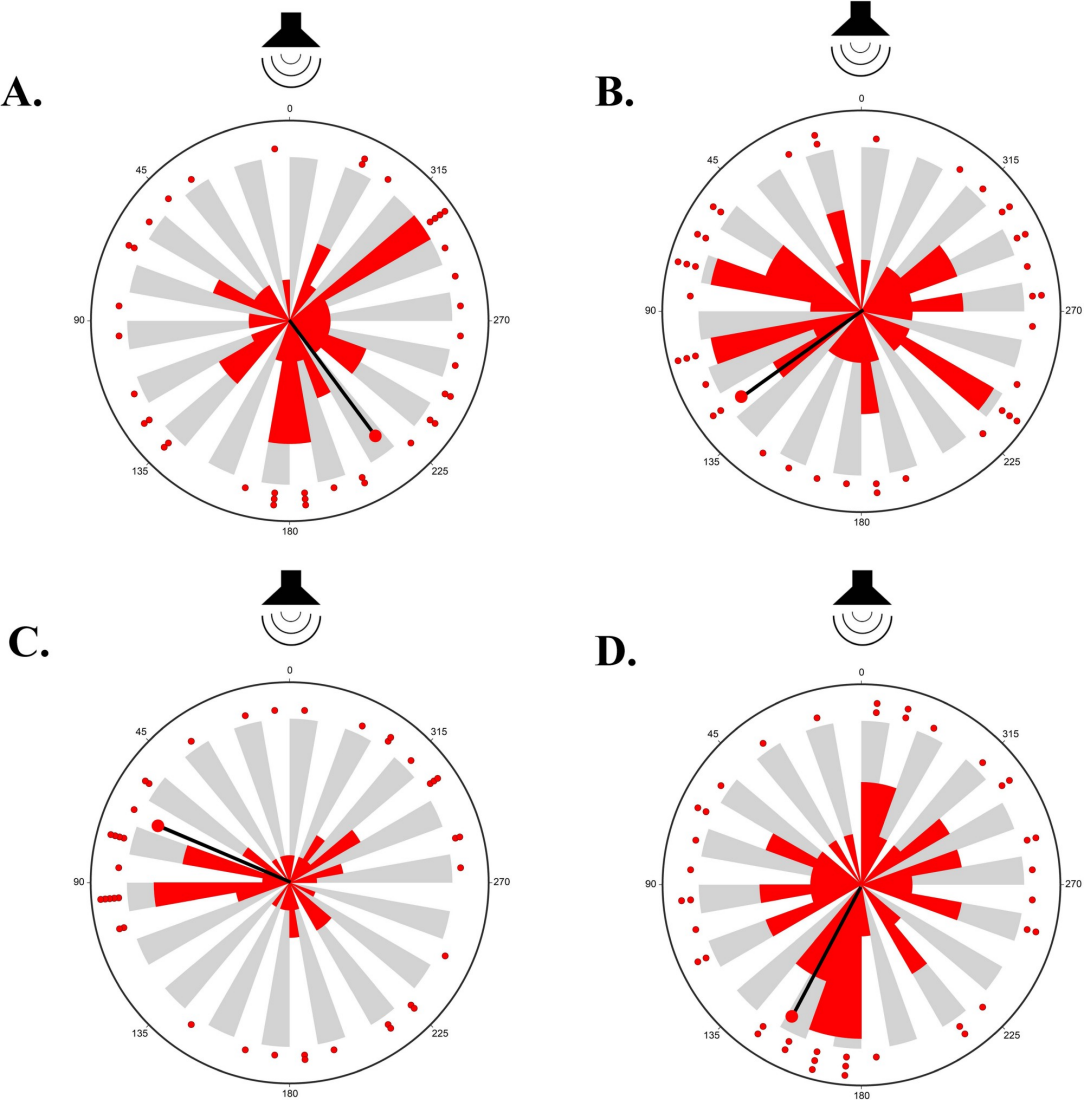
**A)** Orientation of leatherback hatchlings in the presence of no sound in the control trial (N = 41). **B)** Orientation of leatherback hatchlings in the presence of wave sounds (N = 40). **C)** Orientation of leatherback hatchlings in the presence of human conversation sounds (N = 41). **D)** Orientation of leatherback hatchlings in the presence of vehicle traffic sounds (N = 41). For all trials, the speaker was at 0°. Grey and white shading on the inside of each circle corresponds to the 36 bins within the circular arena. Red dots and the corresponding red bar within each grey or white array indicate the number of hatchlings that oriented to each particular bin within the allotted 5-minute trial time. The thin black line capped with a red circle on the inner part of each rose plot indicates the mean orientation angle within each trial group.

In control trials, hatchlings randomly oriented throughout the circle arena (mean angle = 216.8°, p = 0.234, N = 41) (Fig 5A). In the presence of the beach wave sounds (72.0 dB re: 20 μPa), hatchlings exhibited no phonotaxic response and also randomly oriented through the arena (mean angle = 152.1°, p = 0.645, Rayleigh Test, N = 40) (Fig 5B). When compared to turtles in the control group, turtles in the wave sounds group took an average of 18.9 seconds less to reach their final location. There was a significant difference in travel time between the control and wave sounds treatment groups (p = 0.029, Aspin-Welch Unequal-Variance T-Test).

When presented with anthropogenic auditory signals, hatchlings also oriented randomly throughout the circular arena. Specifically, in the presence of human conversation (72.4 dB re: 20 μPa), hatchlings exhibited no phonotaxic response (mean angle = 67.4°, p = 0.554, Rayleigh Test, N = 41) (Fig 5C). Hatchlings in the human conversation group took an average of 1.3 seconds longer than turtles in the control group to reach their final location, however there was not a significant difference in travel time between the control and human conversation treatment groups (p = 0.918. Equal-Variance T-Test). In the presence of traffic noise (71.1 dB re: 20 μPa), hatchlings likewise exhibited no phonotaxic response (mean angle = 125.7°, p = 0.887, Rayleigh Test, N = 41) (Fig 5D). Turtles in the vehicle traffic noise group had an average travel time of 10.0 seconds less than turtles in the control group, however there was not a significant difference in travel time between the control and traffic treatment groups (p = 0.357, Equal-Variance T-Test).

## Discussion

Our results question the potential of hatchlings to use aerial beach wave noise as a sea-finding orientation cue. In the presence of beach wave sounds, hatchlings exhibited no phonotaxic response, however they took less time to orient than hatchlings in the presence of no sound, which may be beneficial as hatchlings would reach the sea faster reducing predation risk. Comparably, in the presence of both human conversation and vehicle traffic noise, hatchlings exhibited no phonotaxic response.

Although we hypothesized that hatchlings would orient toward the wave noise and away from human conversation and vehicle traffic noise, these hypotheses present the beginning of research into the role of sound in sea turtle behavior within their natural environment. Previous studies have been limited to underwater sea turtle responses to unnatural acoustic cues and have not examined the physiological and behavioral responses of sea turtles to sounds in air, or responses to natural sources of environmental sound [27–32]. Our results suggest that the sounds (types and sound pressure levels) we presented to hatchlings did not elicit behavioral responses. Previous studies have shown that the sea turtle ear is most sensitive to low-frequency underwater acoustic stimuli [11, 14, 18]. While we found no phonotaxic behavior amongst the hatchlings in this study, it is important to caveat our findings that other sound types or sound pressure levels may impact sea turtle behavior in air, and sound may still play a role in the biology and behavior of sea turtles underwater. Notably, several studies examining the impacts of unnatural underwater sounds on turtles have shown that sea turtles behaviorally respond to sound. In the presence of both explosions and seismic airguns, sea turtles displayed prominent behavioral changes, including erratic swimming and diving behavior, indicating sensitivity to changes in sound pressure [27–32]. In many of these studies, sea turtles exhibited a negative phonotaxic response within a controlled environment; yet, they demonstrate the distinct potential for sea turtle behavioral and physiological responses to sounds. Additionally, behavioral studies of sea turtle hearing have indicated the capacity for loggerhead sea turtles to learn to respond to acoustics cues to perform a behavior task [36, 37].

It is also possible that aerial sound cues may play an intervening role in sea turtle behavior when paired with other already documented sensory cues. For hatchling orientation, in particular, research has shown that visual and slope cues play a large role in sea-finding [33, 38, 39]. Once they have emerged from their nests, hatchlings orient toward the lowest, brightest horizon, away from dunes and vegetation and toward the broad-open horizon where starlight, moonlight, and sunlight are reflected on the ocean’s surface [33, 39, 40]. When these cues are conflicted by artificial illumination on nesting beaches, hatchlings have been noted to display altered behavior including misorientation and delayed sea-finding that can lead to possible death due to dehydration, exhaustion, or predation [34, 35, 41]. While much focus and research has been dedicated to the role that light and slope play in sea-finding behavior, sound may also play a supporting role. Although no conclusive evidence of phonotaxic behavior was noted in this study, it does not negate the possibility that phonotaxic behavior may exist in hatchlings when coupled with vibration, light, or slope cues. As light and slope have already been demonstrated to play a supreme role in the orientation behavior of hatchlings, it is possible that the lack of light and slope within this study may have negated their orientation abilities altogether. Future studies examining the sea-finding behaviors of hatchling sea turtles should explore the role of aerial acoustic cues when combined with light and slope cues.

Sound may be perceived by hatchlings as a function of both aerial acoustic cues and the accompanying vibrations these sounds produce. Lenhardt (1981) hypothesized that turtles use bone conduction in sound perception by coupling their ear to the substrate [42]. We eliminated vibrations from our study by suspending the speaker off of the ground and arena table. The vibrations associated with beach wave energy, human conversation, and traffic noise dissipate greatly over the beach landscape. It was not possible to replicate the vibrations that hatchlings would have felt from these sounds within the confines of the refuge office. The role that vibration plays in sound perception should be explored in future studies.

Additionally, the sound localization abilities of hatchling sea turtles may explain our lack of observed responses. No published studies exist on localization in sea turtles, though the ability has been observed in captivity. An unpublished study on one sub-adult green sea turtle, conducted behavioral trials where a clicker was played in front of, behind, to the right of, or to the left of the turtle. The turtle’s ability to locate the source of the clicker sound increased significantly over the course of six weeks [43]. While these results show the potential for auditory localization in sea turtles, the study did not report the frequency of the clicker sound played, which hinders comparisons to the frequency range and peak sensitivity of green sea turtle hearing. Moreover, localization abilities in sub-adult sea turtles do not confirm the ability in hatchlings due to the anatomical size differences between the skulls of hatchling and older turtles, specifically in relation to distance between the ears.

Published evidence of localization in other turtle species is presented by Lenhardt (1981). Looking at the semiaquatic turtle, *Chrysemys scripta*, and the terrestrial turtle, *Terrapene carolina major*, Lenhardt (1981) observed behavioral patterns indicative of sound localization. The turtles in Lenhardt (1981) displayed head scanning, as well as retreating and advancing behaviors over a 30 to 90-minute period before making a decision as to where the sound was located [42]. The hatchling turtles in this study, on the other hand, oriented within the circular arena in under 5 minutes, indicating very little time was allocated toward deciding which side of the arena to move toward. Review of the trial videos revealed the hatchlings did not exhibit any head scanning or advancing and retreating behaviors indicative of sound localization in this study. Additional studies should be conducted to determine the extent to which sea turtles can localize sound at various life stages for more conclusive analysis of the role of sound in orientation behaviors.

**Fig 6:**
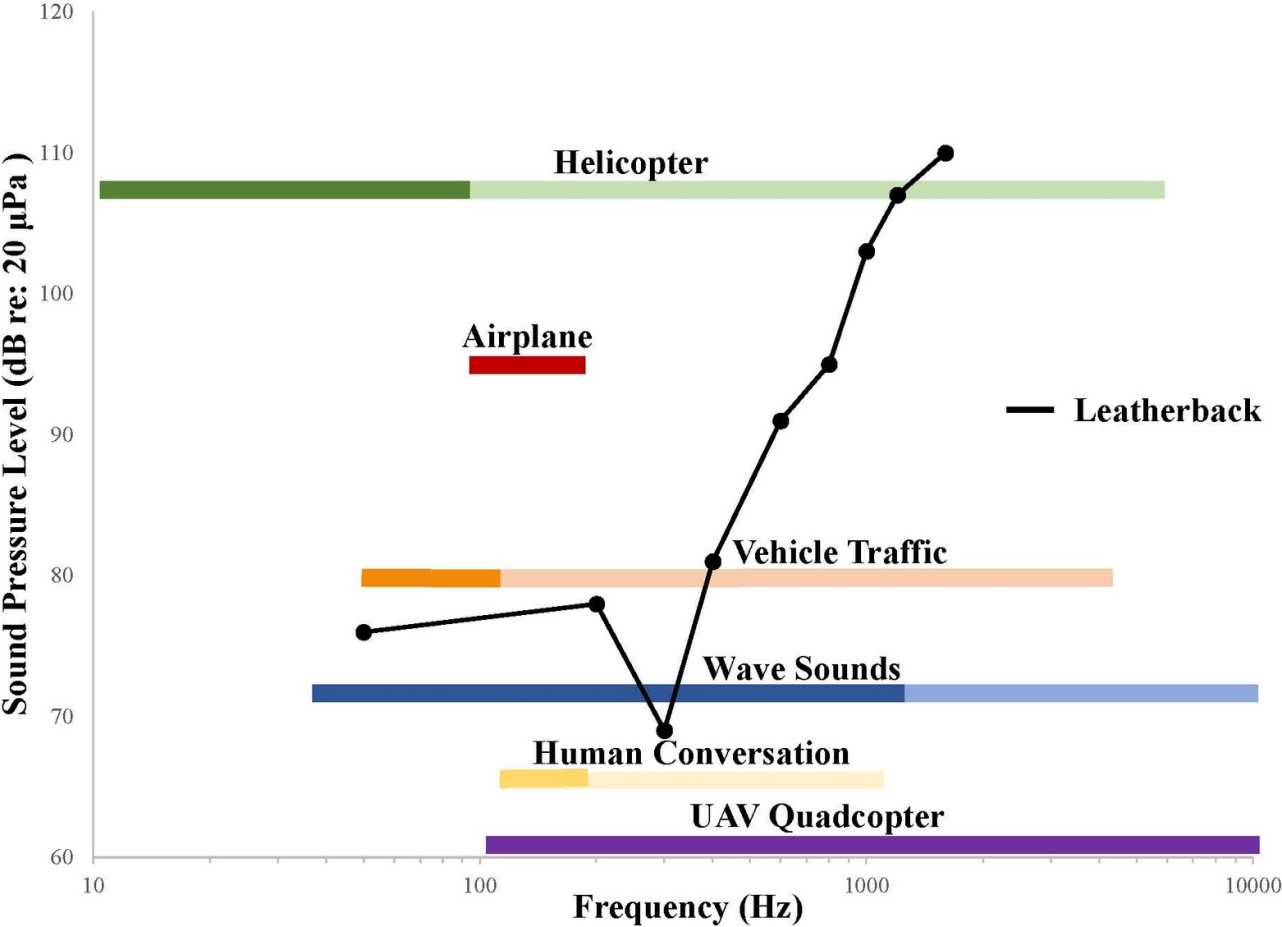
Frequency of aerial sounds (helicopter: 20 and 22, airplane: 19 and 24, vehicle traffic: 21, human conversation: 23, and wave sounds: current study) overlapping with an average hatchling leatherback sea turtle audiogram [11]. The points in the audiogram represent the lowest sound pressure level (SPL) detected and leatherback hatchlings can detect acoustic signals above these SPLs at their associated frequencies. Note that this audiogram presents an average; however, the most sensitive threshold for an individual hatchling recorded in Piniak et al. (2012) was 62 dB re: 20 μPa at 300 Hz. Where available dominant frequency bands of each aerial sound source are depicted with a darker shade of the color representing that sound type.

Finally, while we based the sound pressure level of our three experimental trial sounds on measurements recorded directly on nesting beaches, it is important to note that these levels at the center of the arena occur near previously recorded leatherback hearing thresholds. Auditory evoked potentials (AEPs) of hatchling leatherbacks revealed aerial hearing sensitivity to frequencies between 50 and 1600 Hz, with a maximum sensitivity between 50 and 400 Hz [11, 14]. Piniak et al. (2012) observed the lowest recorded response of leatherback hatchlings to sound at 62 dB re: 20 μPa at 300 Hz [11, 14]. Our three trial signals consisted of wave sounds at 72.0 dB re: 20 μPa, human conversation at 72.4 dB re: 20 μPa and vehicle traffic at 71.1 dB re: 20 μPa at the start location in the center of the arena. As noted previously, the frequency of each of these three trial signals overlaps significantly with the peak hearing range of leatherback sea turtles (Fig 6). Though the sound pressure level of our trial sounds is near leatherback hearing thresholds [11, 14], the sound pressure level of the trial signal increased significantly with distance to the speaker. Directly in front of the speaker, the sound pressure level ranged between 74 and 80 dB re: 20 μPa (Fig 2). We chose not to make any adjustments or increases to the sound pressure level of the trial signals as they would have no longer represented real world conditions and would have prevented any meaningful interpretation of our potential findings. If hatchlings are able to localize sound, they should have been able to perceive an increase or decrease in the sound pressure level as they moved within the arena. However, we noted that rather than exploring the arena and potentially detecting changes in the sound field, most hatchlings made a directional choice and stuck with it when crawling within the arena. This lack of exploration might be explained by a hatchling’s need to get to their final destination (the ocean) quickly in order to reduce predator interactions. Future studies should explore whether our results are consistent with additional aerial acoustic sounds present in the beach environment, such as predator vocalizations and noise produced by airplanes, helicopters, drones, and ATVs. Additionally, human conversation and vehicle traffic should be explored at higher sound pressure levels which may be present in beach locations with greater populations and busier roads.

## Conclusion

The results of this study indicate the need for further auditory orientation experiments to better understand hatchling behavioral responses to aerial environmental acoustic cues. While our results suggest that sound does not appear to play a role in sea-finding behavior, we caution that a range of other variables must be examined before sound is determined not to have an effect on sea turtles on land. As anthropogenic sound sources continue to increase in frequency and intensity, it remains critical to better understand how sea turtles use acoustic cues in order to address the possible impacts of anthropogenic noise, especially as physiological and behavioral impacts have been observed in a variety of other marine including marine mammals and fish [2, 5, 6, 8–10, 44]. Future studies should specifically focus on the sound localization ability of sea turtles in various life stages and on the role that sound may play when combined with other sensory and environmental cues. A better understanding of the biological significance of acoustic cues for sea turtles may lead to the creation of more effective conservation and management plans.

## Supporting information

S1 Table Orientation of hatchlings by speaker location and magnetic cardinal direction. (DOCX)
