# Supplementary material for: Influence of environmental and anthropogenic acoustic cues in sea-finding of hatchling leatherback (Dermochelys coriacea) sea turtles
Source: PLoS One. 2021 Jul 1;16(7):e0253770. doi: 10.1371/journal.pone.0253770 (PMC8248618; doi:10.1371/journal.pone.0253770)
Supplement: S1 Table — (DOCX) [file pone.0253770.s001.docx]

**S1 Table. Orientation of hatchlings by speaker location and magnetic cardinal direction.**

| **Trial Group** | **Speaker Location** | **Mean Angle of Orientation** | **Rayleigh Test** |
| --- | --- | --- | --- |
| No Sound (Control) | 0°, South | 213.35° | Z= 0.245, p=0.791 |
| No Sound (Control) | 90°, East | 329.71° | Z=2.543, p=0.076 |
| No Sound (Control) | 180°, North | 24.432° | Z=0.669, p=0.522 |
| No Sound (Control) | 270°, West | 343.81° | Z=0.718, p=0.501 |
| Wave Sounds | 0°, South | 183.62° | Z=0.936, p=.0403 |
| Wave Sounds | 90°, East | 34.02° | Z=0.083, p=0.924 |
| Wave Sounds | 180°, North | 346.96° | Z=0.205, p=.823 |
| Wave Sounds | 270°, West | 347.39° | Z=0.754, p=0.482 |
| Human Conversation | 0°, South | 56.73° | Z=2.089, p=0.123 |
| Human Conversation | 90°, East | 345.10° | Z=0.972, p=0.388 |
| Human Conversation | 180°, North | 345.59° | Z=0.78, p=0.47 |
| Human Conversation | 270°, West | 321.92° | Z=1.909, p=0.149 |
| Traffic Noise | 0°, South | 174.78° | Z=0.425, p=0.665 |
| Traffic Noise | 90°, East | 260.91° | Z=0.722, p=0.497 |
| Traffic Noise | 180°, North | 196.09° | Z=0.316, p=0.739 |
| Traffic Noise | 270°, West | 285° | Z=0.419, p=0.668 |
